# Real-world Roche Elecsys^®^ and Fujirebio Lumipulse^®^ inter-platform comparability and clinical validation of plasma neurofilament light chain in multiple sclerosis

**DOI:** 10.1007/s00415-026-14068-6

**Published:** 2026-08-25

**Authors:** Vincenzo Criscuolo, Valerio Nicolella, Carmela Polito, Rosa Sirica, Mariano Fiorenza, Domenica Sorvillo, Ilaria Delle Cave, Antonio Carotenuto, Maria Petracca, Roberta Lanzillo, Giuseppe Castaldo, Vincenzo Brescia Morra, Daniela Terracciano, Marcello Moccia

**Affiliations:** 1 Department of Neuroscience, Reproductive Science and Odontostomatology, Federico II University of Naples, Naples, Italy; 2 Department of Precision Medicine in Medical, Surgical and Critical Care, University of Palermo, Palermo, Italy; 3 Department of Translational Medical Sciences, Federico II University of Naples, Naples, Italy; 4 Multiple Sclerosis Unit, Policlinico Federico II University Hospital, Naples, Italy; 5 Department of Human Neuroscience, Sapienza University of Rome, Rome, Italy; 6 Department of Molecular Medicine and Medical Biotechnology, Federico II University of Naples, Naples, Italy; 7 Centre for Advanced Biotechnology (CEINGE), Naples, Italy

**Keywords:** Multiple Sclerosis, Neurofilament light chain, Platform, Lumipulse, Atellica

## Abstract

**Background:**

Neurofilament light chain (NfL) reflects neuro-axonal injury in multiple sclerosis (MS), but method-dependent bias limits interchangeability across automated platforms. We assessed agreement between Roche Elecsys^®^ and Fujirebio Lumipulse^®^, evaluated Simoa-referenced harmonization, validated Elecsys^®^ clinical associations, and derived directional IVD-to-IVD conversion models between Elecsys^®^ and Lumipulse^®^.

**Methods:**

In this retrospective real-world cohort (*N*=253), fasting EDTA plasma was analyzed in singlicate on Elecsys^®^ and Lumipulse^®^. Clinical associations of Elecsys^®^ pNfL were tested using multivariable linear models, including age, sex, disease duration, EDSS, cardiovascular comorbidities, smoking, and disease course, with sensitivity analyses including BMI.

**Results:**

On the raw scale, platforms were strongly correlated but showed poor agreement (Coeff 6.91, 95% CI 6.44–7.38; *r*=0.879; CCC=0.107, 95% CI 0.090–0.123). After conversion of both assay outputs to published Simoa-equivalent values, alignment improved (Coeff 1.03, 95% CI 0.96–1.10). We derived a directional Elecsys^®^-to-Lumipulse^®^ Passing–Bablok conversion (Lumipulse = 1.97 + 11.171 × Elecsys). Higher Elecsys^®^ pNfL was associated with disability (EDSS: Coeff 0.302, 95% CI 0.122–0.481; with BMI: Coeff 0.335, 95% CI 0.119–0.551) and was lower in treated versus untreated patients.

**Conclusions:**

Elecsys^®^ and Lumipulse^®^ are not interchangeable on the raw pg/mL scale though conversion-based approaches can improve cross-platform alignment within the observed analytical range. Elecsys^®^ pNfL retained expected clinical associations, supporting routine MS use. Findings support cautious conversion during platform transitions in clinics.

## Background

Neurofilament light chain (NfL) is a structural protein of the neuronal cytoskeleton released following neuro-axonal injury and measurable in both cerebrospinal fluid and blood. As such, blood NfL (bNfL) has emerged as a quantitative biomarker in multiple sclerosis (MS) and other neurological diseases, reflecting the extent of neuro-axonal damage independently of the underlying pathological mechanism [1]. Recent consensus guidance recommends the integration of bNfL into routine MS care, with careful consideration of pre-analytical, analytical, and interpretative factors [2].

Despite the increasing availability of fully automated platforms for bNfL measurement, there are limited real-world head-to-head data assessing their interchangeability and clinical comparability. Existing evidence suggests that, while correlations across platforms are typically high, systematic method-dependent differences in absolute values may preclude direct interchangeability, thereby requiring assay-specific interpretation and robust calibration strategies [2–7].

Among automated immunoassays, both Roche Elecsys^®^ (cobas e801) and Fujirebio Lumipulse^®^ have been granted CE-IVD (in vitro diagnostics) and have shown clinical applicability in MS [8, 9] [10]. Lumipulse^®^ has been validated in real-world and multicenter cohorts, reproducing expected associations between bNfL levels and MS activity and disability [8, 9]. Elecsys^®^ has also shown significant clinical associations [10] but seems to yield systematically lower absolute values compared with Quanterix Simoa^®^ [3;6;11]. Notably, Simoa has been extensively used to derive age- and body mass index (BMI)-adjusted percentiles and Z-scores, which support individualized risk stratification and longitudinal disease monitoring [11–13].

The overarching aim of this study was to determine whether Roche Elecsys^®^ and Fujirebio Lumipulse^®^ plasma NfL results can be interpreted coherently in routine MS care despite platform-dependent differences. To address this question, we assessed raw-scale agreement between the two platforms, explored their alignment on a common Simoa-referenced scale using published conversion approaches, derived directional IVD-to-IVD conversion models between Elecsys^®^ and Lumipulse^®^, and evaluated whether Elecsys^®^ pNfL retained expected clinical associations in a real-world MS population. The clinical validation component focused on Elecsys^®^ pNfL because the clinical utility of Lumipulse^®^ pNfL in MS had already been evaluated in previous real-world work from our group [8].

## Methods

### Study design and population

This is a retrospective analysis of prospectively collected data conducted at the MS Clinical Unit of the Federico II Policlinico University Hospital (Naples, Italy). This study was approved by the Campania 3 Ethics Committee (protocol 158/25). All participants provided written informed consent for the use of anonymized data in compliance with GDPR EU 2016/679. This study was performed in accordance with Good Clinical Practice and the Declaration of Helsinki.

We consecutively enrolled adults (age >18 years) with a diagnosis of MS who attended scheduled neurological consultations between September 2023 and October 2025, irrespective of age, disease course, expanded disability status scale (EDSS), or treatment status. Patients were asked to participate to the study at their scheduled neurological consultation and blood drawn for plasma neurofilament light chain (pNfL) testing.

### Demographic and clinical variables

At baseline (time of blood draw for pNfL measurements), we collected the following variables: age, sex, smoking (ever or never smoker), height and weight (and derived BMI), cardiovascular comorbidities (high blood pressure, high cholesterol, diabetes, atrial fibrillation, stroke, coronary disease, and/or related medications), MS disease duration (time from reported onset to assessment), EDSS, descriptor of disease progression (relapsing, progressive), current disease-modifying treatment (DMT), and duration of current DMT. For statistical purposes, DMTs were categorized into monoclonal antibodies (alemtuzumab, natalizumab, ocrelizumab, and ofatumumab), oral therapies (cladribine, dimethyl fumarate, fingolimod, siponimod, ozanimod, ponesimod and teriflunomide), and injectables (peg-interferon beta, interferon beta and glatiramer acetate) [14].

We assessed clinical relapses (defined as new, worsening, or recurrent neurological symptoms lasting at least 24 h in the absence of infection, fever, or adverse reaction to prescribed medication), MRI activity (defined as new or enlarging T2 lesions and/or T1 gadolinium-enhancing lesions, as documented by a neuroradiologist on the official radiology report) and EDSS progression (defined as an increase of 1.5 points if baseline EDSS was 0, 1.0 point if baseline EDSS was between 1.0 and 5.5, or 0.5 points if baseline EDSS was >5.5, progression was confirmed over routine follow-up) [15]. To summarize activity composites, relapses and active MRI lesions were jointly combined to define evidence of disease activity 2 (EDA-2), and EDA-2 together with EDSS progression defined evidence of disease activity 3 (EDA-3), as conventionally done in NfL studies [3]. EDA-2 and EDA-3 were assessed in the 6 months preceding baseline (time of blood draw) and were then retrieved for the subsequent clinical visits.

### Neurofilament light chain measurements

Fasting blood samples were obtained on the same day as the clinical assessments. Blood was collected by venipuncture into BD Vacutainer^™^ tubes containing ethylene-diamine-tetra-acetic acid (EDTA) as anticoagulant. EDTA plasma was used as the primary matrix; samples were processed within 3 h of collection (typically within 2 h) and centrifuged at 2000 × g for 10 min at room temperature to obtain plasma. After centrifugation, EDTA plasma was immediately separated and aliquoted into multiple polypropylene tubes, with distinct aliquots dedicated to each analytical platform. All samples were stored at −80 °C until analysis and were not measured as fresh, never-frozen specimens on the day of collection. For each platform, the dedicated aliquot was thawed only once immediately before measurement. No sample underwent repeated freeze–thaw cycles.

Plasma NfL (pNfL, pg/mL) concentrations were measured in singlicate, reflecting routine clinical laboratory practice, using two fully automated immunoassays: Roche Elecsys^®^ NfL on the cobas pro e801 module (cobas e801; two-step electrochemiluminescence immunoassay, ECLIA; Roche Diagnostics, IN, USA) and Fujirebio Lumipulse^®^ G NfL Blood on the LUMIPULSE^®^ G600 (two-step chemiluminescent enzyme immunoassay, CLEIA; Fujirebio, Tokyo, Japan). Paired Elecsys^®^ and Lumipulse^®^ measurements were performed between June 2025 and January 2026 over three analytical sessions. Manufacturer-provided internal quality controls were run at the beginning of each analytical session according to the manufacturers’ instructions and routine laboratory quality assurance procedures. As this was a retrospective real-world study conducted within routine laboratory activity, cumulative intra- and inter-session CVs were not calculated. However, internal quality-control results were reviewed for all analytical sessions and were consistently within the predefined acceptable performance limits for both Elecsys^®^ NfL and Lumipulse^®^ G NfL Blood assays.

For analytical comparison, all Elecsys^®^ NfL measurements were performed using a single reagent lot, and all Lumipulse^®^ G NfL Blood measurements were performed using a single reagent lot.

All analyses were conducted using EDTA plasma, and no serum–plasma cross-matrix conversions were applied [16]. Absolute pNfL concentrations (pg/mL) were used for primary analyses. In addition, to enable comparison on a common reference scale, both Lumipulse^®^ and Elecsys^®^ measurements were converted to Simoa-equivalent values based on previously published calibration approaches [3, 6, 17]. This Simoa-referenced approach was used as an exploratory harmonization layer to compare both platforms on a common literature-based scale, rather than as a proposed clinical replacement for assay-specific interpretation on Elecsys^®^ or Lumipulse^®^ scales.

### Statistical analyses

Continuous variables were summarized as mean (with standard deviation) or median (with range), and categorical variables as counts (with percentages).

To compare Roche Elecsys^®^ NfL and Fujirebio Lumipulse^®^ G NfL Blood assays, we used a univariable linear regression model to evaluate the linear association between pNfL measurements obtained with the two assays on the raw pg/mL scale. Agreement between Elecsys^®^ and Lumipulse^®^ was evaluated using Lin’s concordance correlation coefficient, Pearson correlation coefficient, and Bland–Altman limits of agreement [3, 6]. In addition, we derived directional IVD-to-IVD conversion models using paired Elecsys^®^ and Lumipulse^®^ measurements within the analytical ranges observed in the study. Passing–Bablok regression was used to derive the Elecsys^®^-to-Lumipulse^®^ conversion equation. As an additional IVD-to-IVD sensitivity analysis, Elecsys^®^ pNfL values were transformed into Lumipulse^®^-equivalent concentrations (Elecsys^®^-derived Lumipulse^®^ values) using the study-derived conversion equation, and the relationship between measured Lumipulse^®^ pNfL and Lumipulse-equivalent values derived from Elecsys^®^ was assessed using linear regression. To provide an additional clinically oriented comparison of classification across scales, we applied the published age-stratified Lumipulse^®^ cut-offs (18–50 years, 51–68 years, ≥69 years) to both the measured Lumipulse^®^ concentrations and the Elecsys^®^-derived Lumipulse^®^-equivalent values obtained through the study conversion equation [18]. Classification agreement was assessed descriptively by comparing, within each age group, the proportion of individuals falling below or above the Lumipulse^®^ cut-off on the measured scale versus the Elecsys^®^-derived Lumipulse^®^-equivalent scale. Results were reported as frequency distributions with row and column percentages within each age group.

As a sensitivity analysis of cross-platform alignment on a common reference scale, both Elecsys^®^ and Lumipulse^®^ measurements were converted to Simoa-equivalent values (Elecsys^®^-derived Simoa values and Lumipulse^®^-derived Simoa values) using published conversion approaches [3, 6, 17]. The linear association between the Simoa-equivalent values derived from the two platforms was then assessed using a linear regression model.

Clinical association analyses focused on Elecsys^®^ pNfL to assess the clinical interpretability of Elecsys^®^ measurements in routine MS care. As the clinical utility of Lumipulse^®^ pNfL has already been evaluated by our group, these analyses were not designed as a head-to-head comparison of the clinical performance of Elecsys^®^ and Lumipulse^®^, but rather to confirm that Elecsys^®^ pNfL retained expected associations with key clinical variables [8]. To evaluate the clinical utility of pNfL measured with Elecsys^®^, we used different univariable linear regression models including each demographic and clinical variable, in turn, as independent variable (age, sex, smoking, presence of cardiovascular comorbidities, MS disease duration, descriptor of disease progression, EDSS, EDA-3 in the previous 6 months, subsequent EDA-3, current DMT group, and DMT duration), and raw Elecsys^®^ pNfL values as dependent variable. Then, the same models were run including the full set of covariates: age, sex, disease duration, EDSS, presence of cardiovascular comorbidities, smoking, and descriptor of disease progression. Secondary models analyzed age- and BMI-adjusted Z-scores/percentiles to leverage age-normalized adjustment [12, 13]. Endpoints during follow-up (relapse, MRI activity, disability progression) were uncommon and were preliminarily explored as binary predictors in logistic models; however, these models were not retained in the final workflow due to the low number of observations [3].

Statistical analyses were performed using Stata 15.0 (StataCorp, College Station, TX, USA) and R version 4.6.0 (R Foundation for Statistical Computing, Vienna, Austria). Results were considered statistically significant if p < 0.05. Data visualizations and figures were generated using Stata 15.0 and R version 4.6.0, with support from the internal UNINA Copilot AI system under the supervision of the authors.

## Results

We included 253 people with MS. Mean age was 46.5 ± 13.4 years; mean disease duration 16.3 ± 11.3 years; median EDSS 3.0 (range 1.0–7.5). Baseline characteristics are summarized in Table 1.

**Table 1:** Baseline characteristics of the population

	*N*=253
Age, years	46.5 ± 13.4
Female, *n* (%)	175 (69.2%)
Disease duration, years	16.3 ± 11.3
EDSS, median (range)	3.0 (1.0–7.5)
BMI, kg/m^2^ (mean ± SD)	25.7 (± 3.9)
Estimated blood volume, mL (mean)	2663 (± 570)
Smoking (ever), *n* (%)	54 (21.3%)
Cardiovascular comorbidity, *n* (%)	81 (32.0%)
Disease course % Relapsing/Progressive, *n* (%)	179 (70.8)/74 (29.2)
DMT, untreated, *n* (%)	6 (2.37%)
Oral DMTs, *n* (%)	114 (45.06%)
Monoclonal antibodies *n* (%)	131 (51.78%)
Injectable DMTs *n* (%)	2 (0.79%)
EDA‑2 in prior 6 months, *n* (%)	27 (10.8%)
MRI activity (pre‑baseline), *n* (%)	9 (3.56%)
Relapse (pre‑baseline), *n* (%)	2 (0.80%)
EDSS progression (pre‑baseline), *n* (%)	19 (7.60%)
Lumipulse pNfL, pg/mL	14.02 ± 12.07 (range 2.75–96.48)
Elecsys pNfL, pg/mL	1.50 ± 1.54 (range 0.30–14.40)

### Assay comparability

**Fig. 1:**
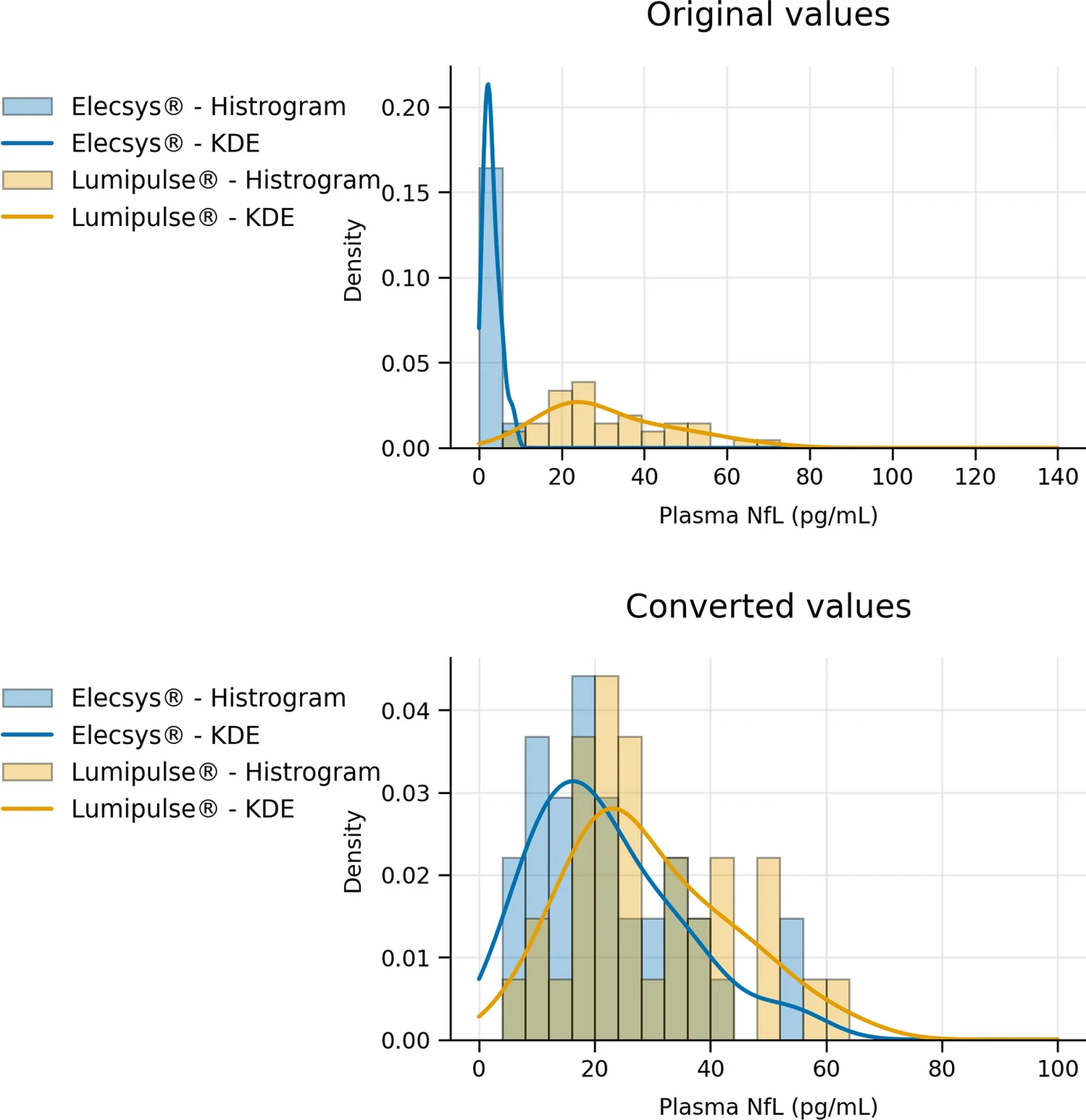
Distribution of plasma NfL on raw and Simoa-equivalent scales. **A** Original raw values (pg/mL) for Elecsys^®^ and Lumipulse^®^, shown as histograms with kernel density estimation curves. **B** Simoa-equivalent values for the same samples after application of published conversion approaches, showing improved distributional alignment on a common literature-based reference scale. Simoa-equivalent values were obtained using the following published formulas: Simoa-equivalent NfL = 6.31 × [Elecsys^®^ NfL] + 2.33 and Simoa-equivalent NfL = 0.94 × [Lumipulse^®^ G NfL Blood] + 1.30 [3]

Mean Lumipulse^®^ pNfL was 14.02 ± 12.07 pg/mL (range 2.75–96.48), whereas mean Elecsys^®^ pNfL was 1.50 ± 1.54 pg/mL (range 0.30–14.40). Distributions on the raw pg/mL scale were right-skewed and differed substantially between platforms, while alignment improved on the Simoa-equivalent scale (Fig. 1).

**Fig. 2:**
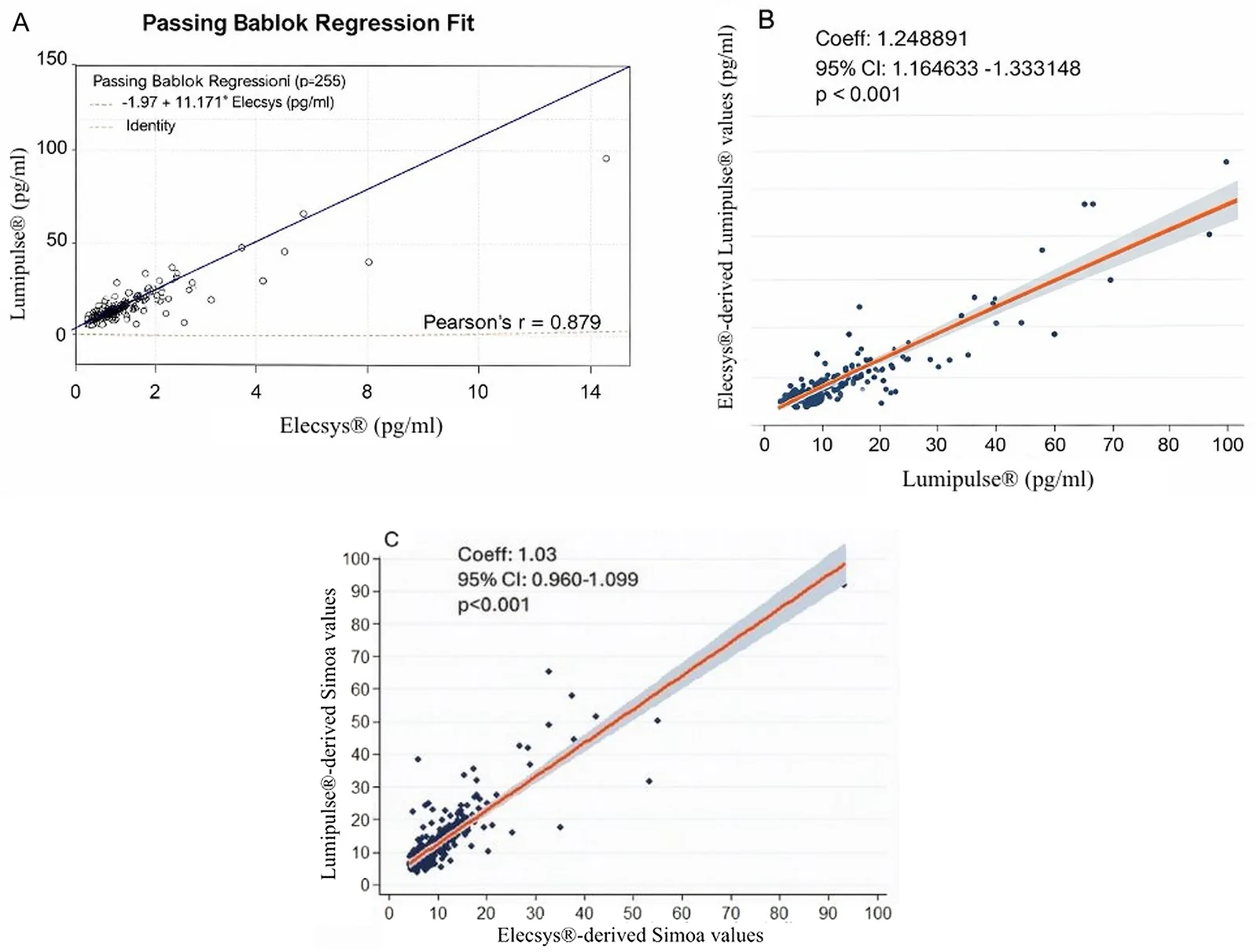
Plasma NfL method comparison and IVD-to-IVD alignment between Lumipulse^®^ and Elecsys^®^. **A** Passing–Bablok regression comparing Lumipulse^®^ pNfL values with Elecsys^®^ pNfL values. The study-derived Elecsys^®^-to-Lumipulse^®^ conversion equation is displayed in the panel. This IVD-to-IVD conversion was derived within the observed analytical range and should not be extrapolated or interpreted as evidence of full interchangeability. **B** Linear regression comparing measured Lumipulse^®^ pNfL values with Elecsys^®^-derived Lumipulse^®^ values, showing strong IVD-to-IVD alignment (Coeff 1.249, 95% CI 1.165–1.333; *p*<0.001). This sensitivity analysis complements the Passing–Bablok conversion shown in (**A**) and should be interpreted within the observed analytical range. **C** Simoa-equivalent comparison: Lumipulse^®^-derived Simoa pNfL values versus Elecsys^®^-derived Simoa pNfL values after application of published conversion approaches, showing improved cross-platform alignment (Coeff 1.03, 95% CI 0.96–1.10; *p*<0.001)

On the raw scale, Lumipulse^®^ and Elecsys^®^ measurements showed a strong linear association (Coeff 6.91, 95% CI 6.44–7.38; *r*=0.879, *p*<0.001), but poor agreement. Passing–Bablok regression analysis demonstrated a significant relationship between Elecsys^®^ and Lumipulse^®^ pNfL measurements and was used to derive the study-specific Elecsys^®^-to-Lumipulse^®^ conversion equation: Lumipulse pNfL = 1.97 + 11.171 × Elecsys pNfL (Fig. 2A). Lin’s concordance correlation coefficient was low (ρc=0.107, 95% CI 0.090–0.123; *p*<0.001), and the bias correction factor (C_b=0.121) indicated substantial deviation from the line of identity, reflecting systematic differences in both scale and location between the two methods.

Bland–Altman analysis confirmed this lack of agreement, showing a mean bias of 12.52 pg/mL (Lumipulse^®^−Elecsys^®^), with wide limits of agreement (− 8.54 to 33.59 pg/mL). Additional analyses (reduced major axis coeff 7.861; Bradley–Blackwood test *p*<0.001) further supported significant differences in both means and variances across platforms.

Given this systematic method-dependent bias, we derived directional IVD-to-IVD conversion models to support cautious cross-platform interpretation within the observed analytical range: Lumipulse pNfL = 1.97 + 11.171 × Elecsys pNfL.

In an additional IVD-to-IVD sensitivity analysis, linear regression comparing measured Lumipulse^®^ pNfL with Lumipulse-equivalent values derived from Elecsys^®^ showed a strong relationship (Coeff 1.249, 95% CI 1.165–1.333; *p*<0.001) as shown in Fig. 2B. Table 2 reports the age-stratified high/low classification of both measured Lumipulse^®^ values and Lumipulse-equivalent values derived from Elecsys^®^, based on the corresponding published Lumipulse^®^ cut-offs.

**Table 2:** Age-stratified high/low classification agreement between measured Lumipulse^®^ values and Lumipulse-equivalent values derived from Elecsys^®^

Age Group		Lumipulse^®^ cut-off (Hanin and colleagues, 2026)	Lumipulse^®^ values, *n* (%)		Total
			Below cut-off	Above cut-off	
18–50 years	Lumipulse^®^-equivalent values derived from Elecsys^®^, *n* (%)	Below cut-off	124 (96.88%)	4 (3.12%)	128 (50.59%)
		Above cut-off	3 (20.00%)	12 (80.00%)	15 (5.93%)
					143 (56.52%)
51–68 years		Below cut-off	75 (98.68%)	1 (1.32%)	76 (30.04%)
		Above cut-off	4 (25.00%)	12 (75.00%)	16 (6.32%)
					92 (36.36%)
Above 69 years		Below cut-off	18 (100%)	0 (0.00%)	18 (7.11%
		Above cut-off	0 (0.00%)	0 (0.00%)	0
					18 (7.11%)
Total			224 (88.54%)	29 (11.46%)	253 (100%)

When values were expressed on the Simoa-equivalent scale, the relationship between platforms approached a near-identity line (Coeff 1.03, 95% CI 0.96–1.10; *p*<0.001), indicating improved cross-platform alignment (Fig. 2C).

### Clinical utility

**Fig. 3:**
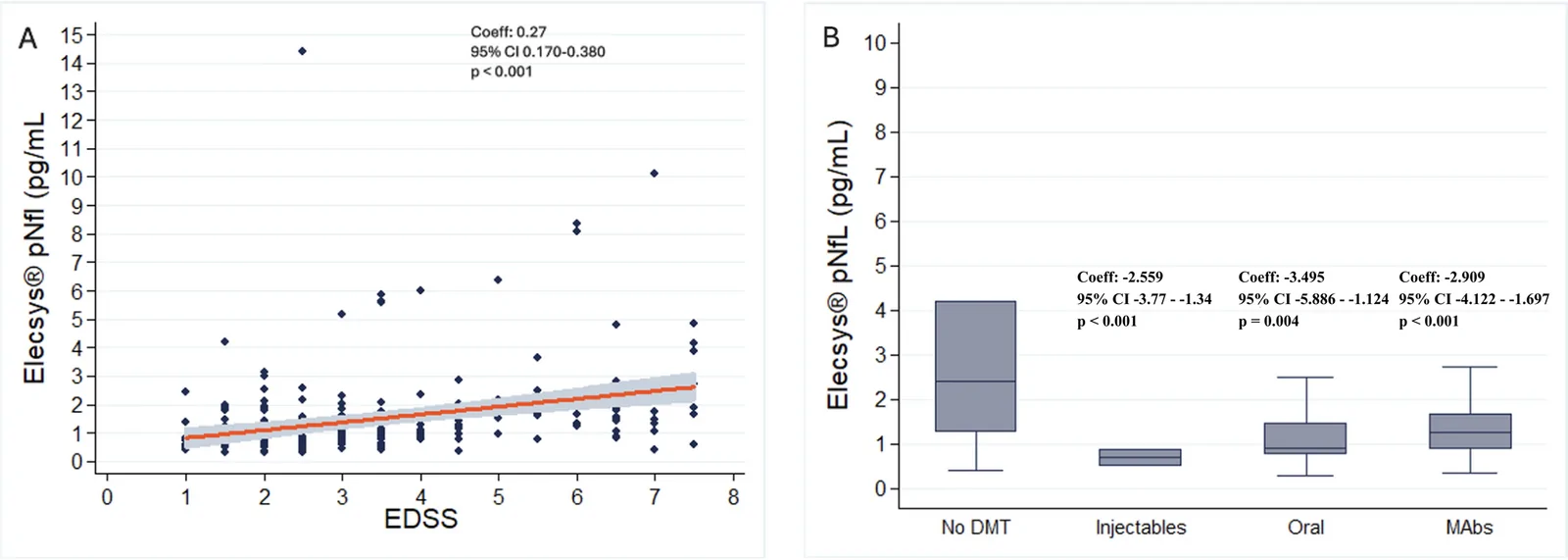
Elecsys^®^ pNfL vs EDSS (unadjusted). Scatterplot with OLS fit and 95% CI band; coeff 0.275 (95% CI 0.170–0.380; *p*<0.001). Boxplots for No DMT, Injectables, Oral, and MAbs. Unadjusted differences vs No DMT: Oral coeff=−3.495 (95% CI −5.866 to −1.124; *p*=0.004); MAbs coeff=−2.909 (95% CI −4.122 to −1.697; *p*<0.001); Injectables coeff=−2.559 (95% CI −3.775 to −1.343; *p*<0.001)

In univariable models, higher Elecsys^®^ pNfL was associated with older age (Coeff= 0.030, 95% CI 0.017–0.044; *p*<0.001) and greater disability (EDSS) (Coeff= 0.275, 95% CI 0.170–0.380; *p*<0.001) (Fig. 3).

In the multivariable model (age, sex, disease duration, EDSS, cardiovascular comorbidity, smoking, disease course), EDSS remained independently associated (Coeff= 0.302, 95% CI 0.122–0.481; *p* = 0.001), whereas age was not significant (*p* = 0.136). Results were unchanged in models also including BMI (EDSS: Coeff= 0.335, 95% CI 0.119–0.551; *p* = 0.003).

Compared with untreated patients, DMT class showed lower Elecsys^®^ pNfL after adjustment (all treated classes vs reference: *p* < 0.05; in the BMI-adjusted specification, one class had p = 0.025 while the others remained < 0.001) (Table 3) (Fig. 3).

**Table 3:** Elecsys^®^ pNfL and demographic, clinical and treatment correlates

Variable	Unadjusted models				Adjusted models			
	Coeff	95% CI – Lower	95% CI – Upper	*p*	Coeff	95% CI – Lower	95% CI – Upper	*p*
Age (years)	0.030	0.017	0.044	<0.001	0.016	−0.005	0.037	0.136
Sex (male vs. female)	0.127	−0.286	0.539	0.546	0.047	−0.351	0.446	0.815
Cardiovascular comorbidity (yes vs. no)	0.536	0.133	0.939	0.009	0.019	−0.445	0.482	0.936
Smoking (ever vs. never)	–	–	–	–	−0.164	−0.617	0.289	0.477
Disease duration (years)	0.028	0.012	0.045	<0.001	−0.002	−0.024	0.020	0.858
EDSS	0.275	0.170	0.380	<0.001	0.302	0.122	0.481	0.001
DMT duration (years)	0.016	−0.060	0.093	0.675	−0.019	−0.094	0.056	0.624
Disease course (progressive vs. relapsing)	0.524	0.110	0.937	0.013	−0.493	−1.082	0.097	0.101
DMT group: Oral vs. No DMT	−3.495	−5.866	−1.124	0.004	−3.241	−5.515	−0.967	0.005
DMT group: Monoclonal antibodies vs. No DMT	−2.909	−4.122	−1.697	<0.001	−2.796	−3.969	−1.623	<0.001
DMT group: Injectables vs. No DMT	−2.559	−3.775	−1.343	<0.001	−2.700	−3.886	−1.514	<0.001
EDA baseline (yes vs. no)	−0.133	−0.754	0.488	0.674	−0.389	−0.989	0.211	0.202

Coefficients (Coeff), 95% confidence intervals (95% CI), and p values from linear regression models including, in turn, demographic, clinical, and treatment features as independent variables, and pNfL levels as the dependent variable. In the adjusted models, covariates were age, sex, disease duration, EDSS, cardiovascular comorbidities, smoking status, and descriptors of disease progression

## Discussion

In this real-world population with MS (*N*=253), paired plasma NfL measurements confirmed that Elecsys^®^ and Lumipulse^®^ NfL assays are highly correlated but not directly interchangeable [3], while demonstrating expected clinical associations.

On the raw pg/mL scale, substantial method-dependent differences were observed, supporting the need for platform-specific reporting as the current standard of care [2, 3, 8]. Although conversion to a common Simoa-referenced scale improved cross-platform alignment, this approach should be regarded as an exploratory bridge to historical Simoa-based literature and reference models, rather than as a recommendation to report clinical results on a research-use-only scale. Consistent with this methodological framework, the age-stratified classification analysis showed that measured Lumipulse^®^ values and Elecsys^®^-derived Lumipulse-equivalent values yielded broadly concordant high/low classifications, particularly among samples below the Lumipulse^®^ cut-off. Residual discordance was observed mainly among samples classified above the cut-off, reflecting the expected variability introduced by cross-platform conversion [18]. These findings support the use of conversion-based alignment as a transitional interpretative aid, while reinforcing that clinical decision-making should continue to rely on assay-specific IVD reference intervals and locally verified decision limits [7]. Accordingly, we complemented the Simoa-referenced analysis with direct IVD-to-IVD conversion analyses between Elecsys^®^ and Lumipulse^®^, supporting cautious cross-platform interpretation during transitions between automated clinical-grade assays [2, 3, 9, 17, 19, 20]. However, these approaches should be restricted to the observed analytical range, should not be extrapolated or inverted, and should be interpreted as pragmatic tools rather than as evidence of interchangeability [2, 21, 22]. As analytical agreement alone does not guarantee clinical usefulness, univariate and multivariate analyses were performed as a complementary validation step to confirm the clinical interpretability of Elecsys^®^ pNfL in routine MS care [2, 7]. From a clinical perspective, Elecsys^®^ pNfL showed expected associations with disability, with higher EDSS scores corresponding to higher pNfL levels, independently of BMI. Treated patients exhibited lower pNfL concentrations compared with untreated individuals, consistent with known treatment effects [2, 8]. In contrast, no association was observed between baseline pNfL and short-term disease activity (EDA-2/EDA-3), likely reflecting the low event rate and limited statistical power of this dataset. Because Lumipulse^®^ pNfL clinical utility in MS has already been evaluated in prior real-world work from our group [8], the present clinical analyses focused on Elecsys^®^ pNfL to determine whether this platform retained expected associations despite raw-scale non-interchangeability with Lumipulse^®^.

Because this study was cross-sectional, we could not assess the longitudinal performance of NfL measurements. Although single time-point NfL concentrations provide clinically relevant information, longitudinal intra-individual changes have been shown to offer greater sensitivity for detecting disease activity and monitoring disease evolution in MS [24]. Therefore, further longitudinal studies are needed to determine whether the analytical differences observed between platforms may influence the interpretation of NfL trajectories over time [2].

Our findings are consistent with prior evaluations showing that automated NfL assays can be strongly correlated, yet remain affected by substantial platform-dependent bias, with Elecsys yielding systematically lower absolute values and thus limiting raw-scale interchangeability [3, 6, 11]. Building on this established analytical background, our study adds a more MS-focused and clinically oriented real-world perspective by integrating agreement-based interpretation and demonstrating that Elecsys^®^ pNfL retains expected clinical associations (e.g., with disability and treatment status). In this way, we move from documenting cross-platform bias to providing pragmatic elements that support transparent interpretation and cautious harmonization when platforms are used in routine MS care. We adhered to a standardized pre-analytical workflow, including fasting sampling, EDTA plasma as the primary matrix, processing within 3 h, and storage at −80 °C, in line with current recommendations and ongoing efforts toward assay harmonization and commutability [12, 13, 17, 19, 20, 23] Previous cross-platform studies, including Dargvainiene et al. [3], incorporated clinical outcomes to demonstrate that different assays capture a comparable biological signal. In contrast, our study adopts a complementary perspective by focusing on how assay-specific measurements—particularly Elecsys^®^ pNfL—can be interpreted in a real-world MS setting despite limited raw-scale interchangeability. Specifically, we show that Elecsys^®^ pNfL retains meaningful associations with disability and treatment status in an unselected MS population, supporting assay-aware clinical interpretation rather than direct numerical interchange.

This study holds several limitations. Its single-center design and focus on MS may limit generalizability. Measurements were performed in singlicate, reflecting routine clinical practice, although replicate testing could further refine analytical precision. Both assays were performed under routine laboratory quality-assurance procedures, and internal quality-control results remained within predefined acceptable limits throughout the analytical sessions. However, because this retrospective real-world study was not specifically designed as a prospective analytical validation or precision study, cumulative intra- and inter-session CVs were not calculated. Future studies specifically designed for analytical validation should include replicate measurements, predefined precision experiments, and external quality-assessment data to further characterize assay performance across platforms. In addition, the study was not designed to evaluate lot-to-lot variability, as a single reagent lot was used for each analytical platform. Therefore, the proposed conversion equation should be regarded as a pragmatic, study-specific tool to support cautious interpretation during platform transitions, rather than as a universally applicable harmonization equation. Further multicenter studies incorporating multiple reagent lots, commutable reference materials, and independent external validation cohorts are required before such conversion formulas can be broadly implemented across laboratories [17, 19, 20, 23].

In conclusion, absolute NfL concentrations are not directly interchangeable between Elecsys^®^ and Lumipulse^®^ automated platforms despite strong correlation. Nevertheless, blood NfL retains its clinical utility in MS across different analytical systems. Future studies should focus on external validation, longitudinal integration with clinical and imaging data, and the development of standardized reference materials and measurement procedures to enable consistent cross-platform interpretation in clinical practice [25, 26]. Establishing robust harmonization frameworks will be critical to fully translate blood NfL from a promising biomarker to a standardized clinical tool.

## Data Availability

Data are available upon reasonable request to the corresponding author.
